# Evaluación de la prueba Fluorecare de anticuerpos contra la proteína Spike del SARS-CoV-2 en la práctica real

**DOI:** 10.1515/almed-2021-0050

**Published:** 2021-06-14

**Authors:** Gian Luca Salvagno, Gianluca Gianfilippi, Laura Pighi, Simone De Nitto, Brandon M. Henry, Giuseppe Lippi

**Affiliations:** Sección de Bioquímica Clínica, Universidad de Verona, Verona, Italia; Servicio de medicina de laboratorio, Hospital Pederzoli, Peschiera del Garda, Italia; Dirección médica, Hospital Pederzoli, Peschiera del Garda, Italia; The Heart Institute, Cincinnati Children’s Hospital Medical Center, Cincinnati, OH, EE.UU

**Keywords:** COVID-19, SARS-CoV-2, medicina de laboratorio, diagnóstico, inmunoensayo

## Abstract

**Objetivos:**

A la luz de la elevada eficiencia diagnóstica de las pruebas rápidas de detección de antígenos (Ag-RDT) contra el coronavirus de tipo 2 causante del síndrome respiratorio agudo severo (SARS-CoV-2), se realizó un estudio con el fin de evaluar el rendimiento clínico de la prueba de antígenos contra la proteína Spike del SARS-CoV-2 en un escenario real.

**Métodos:**

La población de estudio estaba formada por pacientes que se sometieron a una prueba diagnóstica ordinaria del SARS-Cov-2 en el Hospital Pederzoli de Peschiera del Garda (Verona, Italia). Se tomó una muestra de exudado nasofanríngeo en el momento del ingreso, que se sometió a un análisis molecular (Altona Diagnostics RealStar® SARSCoV-2 RT-PCR Kit) y de antígenos (Prueba Fluorecare de Antígenos contra la proteína Spike del SARS-CoV-2).

**Resultados:**

La población de estudio estaba compuesta por 354 pacientes (edad media, 47±20 años; 195 mujeres, 55,1%), de los cuales 223 (65,8%) obtuvieron un resultado positivo en el análisis molecular. Se observó una correlación significativa entre la prueba Fluorecare y Altona (para los genes *S* y *E*: r=−0,75; p<0,001). El área bajo la curva acumulada en todas las muestras nasofaríngeas fue de 0,68. A un índice S/CO ≥1,0, indicado por el fabricante, la sensibilidad, especifidad y valor predictivo negativo y positivo fueron del 27,5%, 99,2%, 41,5% y 98,5%, respectivamente. La sensibilidad se mostró inversamente proporcional a los valores de Ct, llegando al 66,7% en las muestras con valores medios de Ct <30, al 90,5% en aquellas con valores medios de Ct <25, y hasta al 100% en las muestras con valores medios de Ct <20.

**Conclusiones:**

Dada la modesta sensibilidad y moderado valor predictivo negativo de la prueba de Fluorecare, no se recomienda sustituir con esta prueba el análisis molecular para el diagnóstico de infección por SARS-CoV-2, aunque su adecuada sensibilidad confirma su fiabilidad para el cribado de pacientes con mayor potencial infeccioso.

## Introducción

Ante la enorme presión soportada por los laboratorios diagnósticos a consecuencia de la actual pandemia de la enfermedad por coronavirus 2019 (COVID-19), las pruebas rápidas de antígenos se postulan como una alternativa para la identificación masiva, rápida y eficiente de pacientes con síndrome respiratorio agudo severo provocado por infección por coronavirus tipo 2 (SARS-CoV-2) [1]. No obstante, tal como recientemente han respaldado la Organización Mundial de la Salud (OMS) [2] y el grupo de trabajo sobre COVID-19 de la Federación Internacional de Química Clínica y Medicina de Laboratorio (IFCC) [3], se debe evaluar concienzudamente el rendimiento clínico de cada prueba de detección rápida de antígenos contra SARS-CoV-2 (Ag-RDT), previamente a su introducción en cualquier nivel de la práctica clínica. El objeto de este estudio es evaluar el rendimiento clínico de la prueba de antígenos contra la proteína Spike del SARS-CoV-2 (en adelante, Fluorecare) en un escenario real.

## Materiales y métodos

### Población de estudio

Nuestra población de estudio estaba compuesta por pacientes que se sometieron a las pruebas diagnósticas de SARS-Cov-2 en el Servicio de Medicina de Laboratorio del Hospital Pederzoli (Peschiera del Garda, Verona, Italia) entre el 2 y el 19 de abril de 2021, por encontrarse sintomáticos o haber estado en contracto estrecho con otros pacientes con COVID-19. Se tomó inmediatamente un exudado nasofaríngeo (Virus swab UTM™, Copan, Brescia, Italia) en el momento del ingreso, que se sometió a un análisis molecular y de antígenos.

### Análisis molecular

La prueba de amplificación de ácidos nucleicos de SARS-CoV-2 (NAAT) se realizó con la prueba Altona Diagnostics RealStar^®^ SARSCoV-2 RT-PCR (Altona Diagnostics GmbH, Hamburg, Alemania). Esta reacción en cadena de la polimerasa con transcriptasa inversa (rRT-PCR) consiste en dos amplificaciones y detecciones dirigidas cada una a las secuencias genéticas *E* y *S* del SARS-CoV-2. Para detectar una posible inhibición en la rRT-PCR, incluye como control interno una sonda y un cebador. La prueba se realizó con un sistema de detección Bio-Rad CFX96™ Deep Well Dx Real-Time PCR Detection System (Bio-Rad Laboratories, Hercules, CA, EE.UU). El resultado era positivo si se obtenían valores del umbral de ciclo (Ct) inferiores a 45 para los genes *S* y *E* del SARS-CoV-2.

### Prueba de antígenos

La prueba de antígenos se realizó con la prueba inmunocromatográfica Fluorecare de anticuerpos contra la proteína Spike del SARS-CoV-2 (Microprofit Biotech, Shenzhen, China). Explicado brevemente, se mezcla una muestra nasofaríngea con una solución de anticuerpos contra la proteína Spike del SARS-CoV-2 marcados con fluorocromos. Los inmunocomplejos resultantes migran a través de la membrana nitrocelulosa al área de detección, donde generan una línea roja (en las muestras positivas para la proteína Spike del SARS-CoV-2). Los anticuerpos contra la proteína Spike del SARS-CoV-2 marcados con fluorocromos también migran hacia la ventana de control de calidad, donde se genera otra línea para verificar que la prueba funciona correctamente. La realización de la prueba completa lleva entre 15 y 30 minutos. Para la lectura cuantitativa de fluorescencia se pueden emplear instrumentos en el mismo punto de atención (Fluorecare MF-T1000; Microprofit Biotech, Shenzhen, China), y se refleja como una unidad de medida arbitraria (esto es, índice S/CO). La prueba es positiva si el índice S/CO es ≥1,0. Según el fabricante, los porcentajes de concordancia positivo y negativo usando la RT-PCR como referencia son del 92,2% y el 100%, respectivamente.

### Análisis estadístico

En nuestro estudio, el rendimiento diagnóstico de Fluorecare frente al NAAT, empleado como referencia, se evaluó mediante la correlación de Spearman, construyendo curvas ROC y calculando su sensibilidad, especifidad, valor predictivo negativo (VPN) y valor predictivo positivo (VPP). El análisis estadístico se realizó con el programa Analyse-it (Analyse-it Software Ltd, Leeds, UK). La investigación se realizó como parte de las actividades del laboratorio clínico, empleando muestras ya existentes tomadas para el diagnóstico del SARS-CoV-2 en el centro, por lo que no fue necesario obtener el consentimiento informado del paciente ni aprobación por parte del Comité Ético. Los resultados de todas las pruebas fueron anonimizados previamente a su análisis estadístico. El estudio se realizó de conformidad con los principios de la Declaración de Helsinki, respetando la legislación nacional pertinente.

## Resultados

La población final de estudio estaba compuesta por 354 pacientes (edad media, 47 ± 20 años; 195 mujeres, 55,1%), 223 de los cuales (65,8%) fueron positivos en NAAT (i.e., valores de Ct <45 para los genes *S* y *E* del SARS-CoV-2). Los valores de Ct de las muestras positivas fueron 29,8 ± 7,1 y 30,3 ± 7,0 para los genes S *y* E del SARS-CoV-2, respectivamente. Observamos una correlación notablemente significativa entre los valores de Fluorecare y los valores de Ct evaluables de los genes *S* (r=−0,75; IC95%, entre −0,80 y −0,69; p<0,001) y *E* (r=−0,75; IC95%, entre −0,80 y −0,68; p<0,001).

El rendimiento diagnóstico global de Fluorecare, así como su rendimiento estratificado por valores de Ct de Altona se muestran en la Tabla 1. El área bajo la curva (AUC) acumulada en todas las muestras nasofaríngeas fue de 0,68. Aplicando el punto de corte ≥1,0 para al índice S/CO indicado por el fabricante, la sensibilidad, especifidad, y valores VPN y VPP fueron del 27,5%, 99,2%, 41,5% y 98,5%, respectivamente. El mejor punto de corte para S/CO, calculado a partir de la curva ROC, fue 0,18, asociado con una sensibilidad del 42,9% (IC95%, 36,5–49,5%), una especifidad del 92,6% (IC95%, 86,4–96,5%), un VPP del 45,7% (IC95%, 42,7–48,8%), un VPN del 91,7% (IC 95%, 85,4–95,5%). La sensibilidad aumentaba notablemente con valores bajos de Ct, llegando al 66,7% en las muestras con valores medios de Ct <30, 90,5% en aquellas con valores de Ct medios <25, y hasta el 100% en aquellas con valores medios de Ct <20.

**Tabla 1:** Rendimiento clínico de la prueba Fluorecare de anticuerpos contra la proteína Spike del SARS-CoV-2 estratificado por valores del umbral de ciclo (Ct).

Valores Ct	n	AUC	SEN	ESP	VPN	VPP
Todas las muestras	354	0,68 (IC95%, 0,62–0,73)	27,5% (IC95%, 21,8–33,7%)	99,2% (IC95%, 95,5–100%)	41,5% (IC95%, 39,6–43,5%)	98,5% (IC95%, 90,0–99,8%)
<30	96	–	66,7% (IC95%, 56,3–76,0%)	–	–	–
<25	63	–	90,5% (IC95%, 80,4–96,4%)	–	–	–
<20	36	–	100% (IC95%, 90,3–100%)	–	–	–

AUC, área bajo la curva; SEN, sensibilidad; ESP, especifidad; VPN, valor predictivo negativo; VPP, valor predictivo positivo.

**Figura 1:**
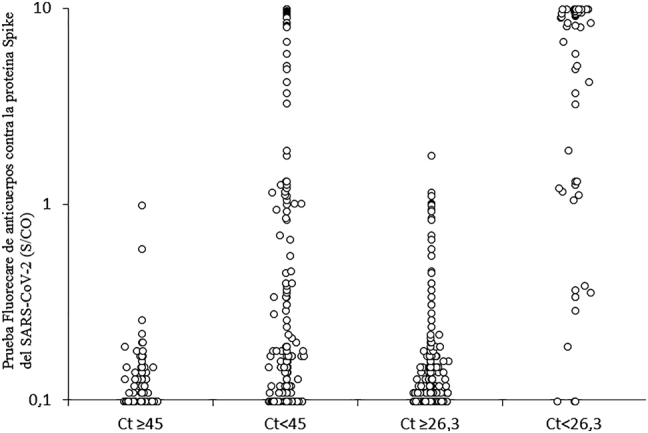
Distribución de los valores obtenidos con Fluorecare aplicando los umbrales de Altona para positividad de la muestra (esto es, valores de Ct <45 de los genes *S* y *E* del SARS-CoV-2) o mayor riesgo de infectividad (esto es, valores de Ct <26,3 de los genes *S* y *E* del SARS-CoV-2). Ct, umbral de ciclo; SARS-CoV-2, coronavirus del síndrome respiratorio agudo grave; S/CO, índice S/CO.

En la Figura 1 se muestra la distribución de los valores obtenidos con Fluorecare con respecto a los umbrales de Altona para positividad de la muestra (esto es, valores de Ct <45 de los genes *S* y *E* del SARS-CoV-2) o mayor riesgo de contagiosidad (valores de Ct <26,3 de los genes *S* y *E* del SARS-CoV-2). El valor medio obtenido con Fluorecare SARS-CoV-2 fue significativamente mayor en las muestras con valores de Ct <45 (n=233; 2,1±3,6 S/CO), frente las muestras con valores de Ct superiores (n=121; 0,1 ± 0,1 S/CO; p<0,001), así como en aquellas con valores de Ct <26,3 (n=68; 6,7 ± 4,0 S/CO), frente a las muestras con valores de Ct superiores a este rango (n=286; 0,2 ± 0,2 S/CO; p<0,001).

## Discusión

El antígeno de SARS-CoV-2, principalmente representado por Ag-RDT, se postula como una posible alternativa que permitiría aliviar la enorme presión que sufren los laboratorios clínicos con motivo de las numerosas pruebas para el diagnóstico de la COVID-19 [1, 4, 5]. Un análisis reciente del Grupo Cochrane de Precisión de Pruebas Diagnósticas para la COVID-19 subraya las aparentes ventajas y posibles inconvenientes de estas técnicas [6]. Con respecto a sus ventajas, estas pruebas son sencillas, las puede realizar cualquier persona que haya recibido una breve instrucción o incluso los propios pacientes; no precisan el uso de analizadores de los laboratorios centrales y ofrecen resultados manejables de una forma rápida. Sin embargo, el rendimiento clínico de los antígenos Ag-RDT sigue siendo considerablemente inferior al del análisis molecular, y su sensibilidad diagnóstica acumulada no logra superar el 60–75% [6]. Se ha demostrado que posee un rendimiento especialmente discreto en muestras con una baja carga viral (valores de Ct ≥25), con una sensibilidad diagnóstica de entre el 30 y el 50%, que aumenta a entre el 91 y el 97% en las muestras con mayor carga viral (valores de Ct <25). Sin embargo, el mismo Grupo Cochrane ha hecho notar la amplia heterogeneidad en el rendimiento diagnóstico de las diferentes pruebas, con sensibilidades que oscilan entre extremadamente bajas (e.g. 12%) y excepcionalmente elevadas (e.g. 90% o incluso superiores). Este escenario incierto ha llevado a la OMS y al Grupo de Trabajo sobre COVID-19 del IFCC a recomendar que cada Ag-RDT se someta a un proceso local de validación, previamente a su introducción en la práctica clínica [2], [3].

Los resultados de este estudio tienen dos implicaciones principales. En primer lugar, la relativamente modesta sensibilidad global y VPN de la prueba Fluorecare de la proteína Spike del SARS-CoV-2 muestra que esta prueba no puede sustituir ni es una alternativa al NAAT para el diagnóstico de la infección por SARS-CoV-2. No obstante, su satisfactoria sensibilidad en las muestras con elevada carga viral sí la hace una prueba adecuada para el cribado de pacientes con un mayor potencial infeccioso. Un estudio reciente publicado por Gniazdowski y col [7]. revela que la probabilidad de obtener un cultivo positivo para SARS-CoV-2 fue <3% en las muestras nasofaríngeas con valores de Ct de Altona <26,2. Aplicando un umbral de Ct similar, Fluorecare mostró una sensibilidad diagnóstica del 90% en nuestra cohorte de pacientes no seleccionados sometidos a una prueba diagnóstica ordinaria de COVID-19, lo que habría permitido identificar a la gran mayoría de los pacientes con una elevada carga viral en el tracto respiratorio superior, que son los que representan un mayor riesgo de expandir el virus en la comunidad (los llamados "supercontagiadores") [8].

Otro aspecto importante a tener en cuenta es que la carga viral nasofaríngea no solo influye en la probabilidad de contagio del SARS-CoV-2 entre individuos, sino que también está relacionada con el riesgo de progresión de COVID-19 a enfermedad grave o crítica y/o mortalidad, tal como ya han demostrado estudios anteriores [9], [10], [11]. De este modo, la identificación rápida y eficaz y/o el seguimiento de los pacientes con COVID-19 con una elevada carga nasofaríngea de SARS-CoV-2 durante el triaje (incluso fuera de los centros sanitarios), así como durante la hospitalización o en el momento del ingreso en la UCI, permitiría establecer un tratamiento más agresivo en el momento oportuno, aumentando así la probabilidad de obtener un buen resultado clínico de esta enfermedad potencialmente mortal.

En conclusión, los hallazgos de este estudio indican que es preferible reservar el uso de esta Ag-RDT para identificar a los pacientes con una mayor carga viral.
